# Fetal Aneuploidy Detection by Cell-Free DNA Sequencing for Multiple Pregnancies and Quality Issues with Vanishing Twins

**DOI:** 10.3390/jcm3030679

**Published:** 2014-06-25

**Authors:** Sebastian Grömminger, Erbil Yagmur, Sanli Erkan, Sándor Nagy, Ulrike Schöck, Joachim Bonnet, Patricia Smerdka, Mathias Ehrich, Rolf-Dieter Wegner, Wera Hofmann, Markus Stumm

**Affiliations:** 1 LifeCodexx AG, Konstanz 78315, Germany; E-Mails: u.schoeck@lifecodexx.com (U.S.); j.bonnet@lifecodexx.com (J.B.); p.smerdka@lifecodexx.com (P.S.); w.hofmann@lifecodexx.com (W.H.); 2 Bahceci IVF Center, Istanbul 34330, Turkey; E-Mail: erbilyagmur@hotmail.com; 3 BioGen Medical Instruments Co. Ltd., Istanbul 34235, Turkey; E-Mail: sanlierkan@biogen.com.tr; 4 Petz Aladár Country Teaching Hospital, Győr 9023, Hungary; E-Mail: nagysandor@gyor.net; 5 Sequenom Center for Molecular Medicine, San Diego, CA 92121, USA; E-Mail: mehrich@sequenom.com; 6 Center for Prenatal Diagnosis and Human Genetics Kudamm-199, Berlin 10719, Germany; E-Mails: wegner@kudamm-199.de (R.-D.W.); stumm@kudamm-199.de (M.S.)

**Keywords:** NIPT, cell-fee fetal DNA, multiple pregnancies, vanishing twin, aneuploidy, trisomy, random massively parallel sequencing

## Abstract

Non-invasive prenatal testing (NIPT) by random massively parallel sequencing of maternal plasma DNA for multiple pregnancies is a promising new option for prenatal care since conventional non-invasive screening for fetal trisomies 21, 18 and 13 has limitations and invasive diagnostic methods bear a higher risk for procedure related fetal losses in the case of multiple gestations compared to singletons. In this study, in a retrospective blinded analysis of stored twin samples, all 16 samples have been determined correctly, with four trisomy 21 positive and 12 trisomy negative samples. In the prospective part of the study, 40 blood samples from women with multiple pregnancies have been analyzed (two triplets and 38 twins), with two correctly identified trisomy 21 cases, confirmed by karyotyping. The remaining 38 samples, including the two triplet pregnancies, had trisomy negative results. However, NIPT is also prone to quality issues in case of multiple gestations: the minimum total amount of cell-free fetal DNA must be higher to reach a comparable sensitivity and vanishing twins may cause results that do not represent the genetics of the living sibling, as described in two case reports.

## 1. Introduction

Since its introduction into clinical practice in 2011, non-invasive prenatal testing (NIPT) for common fetal trisomies by random massively parallel sequencing (rMPS) of cell-free DNA in maternal plasma has been carried out successfully in thousands of singleton pregnancies. At that time, twin and other multiple pregnancies had to be excluded from the clinical practice because of the lack of clinical validation data [1]. Due to an increase in the use of assisted reproductive technologies (ART) over the last decades, multiple gestations have become ever more prevalent. As on the one hand first-trimester screenings in twin pregnancies have a false-positive rate (FPR) that is two to three times higher than that of single pregnancies [2] and on the other hand multiple gestations are prone to complications and bear an elevated risk for procedure related losses after amniocenteses [3], NIPT offers a valuable improvement for this group of pregnant women.

The lower limit for detection of a fetal aneuploidy by rMPS of maternal plasma in single pregnancies has been defined in several studies to be a fetal fraction of 4% [4,5,6,7]. In principle, in monozygous twin pregnancies the fetuses should have concordant karyotypes and, thus, the same lower limit for detection should apply. If a trisomy occurs in a dizygous twin pregnancy, usually only one of the fetuses is affected. It is then crucial that each twin contributes enough cell-free fetal DNA (cffDNA) to discriminate between aneuploid and euploid pregnancies. More and more clinical data are showing that the sensitivity and specificity for the detection of fetal trisomy 21 by NIPT in twin pregnancies—monozygous as well as dizygous—are comparable to those in singleton pregnancies [8,9,10].

A clear limitation for the use of NIPT is the existence of a vanishing twin, which may also occur in pregnancies after the use of ART. Here, we report the results of an NIPT study for the detection of common fetal trisomies in multiple pregnancies, comprising a retrospective and a prospective part, as well as two cases of vanishing twins that occurred in routine clinical practice and caused discordant results.

## 2. Materials and Methods

### 2.1. Subject Enrollment

For the retrospective part of the study, 16 twin samples were provided by Sequenom Inc., USA, from the U.S. Trial No. NCT00877292, as blinded DNA sequencing libraries. The corresponding karyotyping results for the samples were available at Sequenom Inc.

For the prospective part of the study, 40 blood samples from women pregnant with multiple gestations (mono-, di- and trichorionic twin and triplet pregnancies) have been consecutively collected during NIPT laboratory routine for research and development (R&D) purposes between 6 November 2012 and 16 November 2013. Two blood samples came from women pregnant with triplets, the remaining 38 samples came from twin pregnancies. From each pregnant woman carrying a multiple pregnancy, two samples each with 7–10 mL venous blood were collected using Streck cell-free DNA blood collection tubes. The blood samples were shipped to LifeCodexx diagnostics laboratory with a maximum delivery time of 4 days.

### 2.2. Sample Processing and DNA Extraction

Plasma preparation was performed by centrifugation (1600× *g* for 10 min at 4 °C) and plasma separation followed by a second centrifugation step (16,000× *g* for 10 min at 4 °C). Extraction of cfDNA was performed with QIAamp Circulating Nucleic Acid Kit (Qiagen, Hilden, Germany) according to the manufacturer protocol using 3.0–4.0 mL plasma with a final elution volume of 60 μL AVE-buffer.

### 2.3. Quality Control of cffDNA (QuantYfeX)

A measurement of the cell-free fetal DNA in relation to total cell-free DNA is required to determine the fetal fraction as a quality control. From the eluted cell-free DNA 11 μL were digested with the CpG methylation sensitive enzymes Hha1 (0.4 U/μL), HpaII (0.3 U/μL) and BstUI (0.3 U/μL) in a 22 μL reaction using CutSmart™ Buffer (New England Biolabs, Frankfurt am Main, Germany). The reaction was incubated for 60 min at 37 °C and 60 min at 60 °C. For a duplicate measurement, two times 10 μL out of the digestion reaction were used as template DNA for quantitative PCR. For the PCR reaction, a two-fold concentrated PCR master mix (QuantiFast Multiplex PCR Kit, Qiagen) is used in a 25 μL reaction. Primers that span CpG methylation sensitive restriction enzyme sites are used in combination with FAM-labelled probes and primers that do not span the relevant restriction sites are used in combination with VIC-labelled probes (for primer sequences see Appendix Table A1 and for thermocycler profiles see Appendix Table A2). The assay design is based on two marker genes, which are described to be hypomethylated in maternal DNA and hypermethylated in fetal DNA [11,12,13]. Quantitative PCR was performed on a LightCycler 480 II Instrument (Roche, Mannheim, Germany) using serial dilutions of male genomic DNA (Promega, Mannheim, Germany) with known concentrations as standards. The fetal fraction is calculated by relative quantification of signals in the FAM channel *versus* the VIC channel and the total cfDNA amount is calculated by absolute quantification of signals in the VIC channel using LightCycler 480 Software release 1.5.0.

### 2.4. Maternal Plasma DNA Sequencing and Data Analysis

DNA sequencing libraries were prepared using NEBNext Ultra™ DNA Library Prep Kit from Illumina. Libraries were prepared according to the manufacturer protocol automated on a Hamilton STARplus robot. Library quality and quantity were measured using a Bioanalyzer instrument (Agilent, Santa Clara, CA, USA) and a Qubit Fluorometer (Life Technologies, Carlsbad, NM, USA). Based on the library quantification, dilutions and equimolar pools of 12 samples per pool were prepared. The pooled samples were sequenced on one lane of an Illumina v3 flow cell on an Illumina HiSeq2000 sequencer (Illumina, Hayward, CA, USA). Clonal clusters were generated using TruSeq SR Cluster Kit v3-cBot-HS on a cBot Cluster Generation System according to the manufacturer’s protocol. Bioinformatic analysis has been carried out as described before, with *z*-scores ≥3 indicating the presence of a fetal trisomy 21 [14].

Results were reported to the responsible physicians as non-validated R&D results within two weeks after receipt of the blood samples. The results were confirmed by invasive diagnostic methods only in the case of positive test results.

For the retrospective part of the study, the results for the 16 samples were communicated to Sequenom. Subsequently, the samples were unblinded and the NIPT results were compared to the karyotyping results.

## 3. Results and Discussion

### 3.1. Retrospective Study on Stored DNA Libraries

Data from the 16 samples from twin pregnancies provided by Sequenom are summarized in Table 1. Four of the samples had *z*-scores for chromosome 21 higher than the cut-off of 3, indicating a positive result for trisomy 21; the respective *z*-scores for chromosomes 13 and 18 were below the chromosome specific cut-off values (for chromosome 21: 3.0, for chromosome 18: 3.2, for chromosome 13: 3.9). The *z*-scores for chromosomes 21, 18 and 13 of the remaining 12 samples were also below the cut-off values. Following unblinding and comparing the NIPT results to the respective karyotypes, all NIPT results were correct with no false-positive or false-negative results, confirming the robustness of the method also for twin pregnancies.

**Table 1:** Characteristics and NIPT results for the stored DNA libraries.

Sample	Chr13 *z*-score	Chr18 *z*-score	Chr21 *z*-score	Fetal fraction (%)	Gestational age (p.m.)	NIPT result	Fetus A karyotype	Fetus B karyotype	Invasive method
RDLN015823	0.0	0.0	−0.4	37	10 + 6	negative	46,XX	46,XY	CVS
RDLN015835	−0.6	0.9	0.7	35	12 + 6	negative	46,XY	46,XX	CVS
RDLN015916	1.8	1.9	0.8	24	16 + 2	negative	46,XY	46,XY	AC
RDLN016042	0.9	0.5	1.0	23	17 + 4	negative	46,XX	46,XY	AC
RDLN016047	1.3	0.7	−1.6	45	13 + 5	negative	46,XY	46,XY	CVS
RDLN016114	−0.9	−0.2	8.4	29	14 + 4	T21 positive	47,XY,+21	46,XX	CVS
RDLN016116	−0.4	0.7	4.4	20	13 + 4	T21 positive	47,XX,+21	46,XX	CVS
RDLN016450	−0.1	1.4	8.4	31	16 + 0	T21 positive	47,XX,+21	46,XX	AC
RDLN016457	1.0	0.9	−0.3	22	17 + 5	negative	46,XX	46,XY	AC
RDLN016474	0.8	0.3	5.4	16	18 + 4	T21 positive	47,XX,+21	46,XX	AC
RDLN016519	0.2	−1.0	0.2	20	15 + 0	negative	46,XX	46,XX	AC
RDLN016778	0.2	−0.1	−0.1	12	16 + 0	negative	46,XX	46,XX	AC
RDLN017192	−1.2	−0.4	−1.0	13	16 + 1	negative	46,XY	46,XY	AC
RDLN017624	1.0	0.9	0.7	15	17 + 0	negative	46,XY	46,XY	AC
RDLN017641	−1.0	0.3	0.7	8	15 + 2	negative	46,XY	46,XY	AC
RDLN017670	0.8	0.1	−0.9	24	17 + 1	negative	46,XX	46,XX	AC

### 3.2. Prospective Study on Blood Samples from Multiple Pregnancies Collected during Laboratory Routine

Blood samples from multiple pregnancies included 38 twin and two triplet cases; they were collected in pregnancy week 9 + 3 to 23 + 0, with the median at 14 + 2. In 15 cases, conception occurred following ART, including seven cases with intracytoplasmic sperm injection (ICSI) and one case with egg donation. In five cases, spontaneous multiple pregnancy was reported, and from the 20 remaining cases, information about conception is not available. Further characteristics and test results for the blood samples are given in Table 2. There were two positive test results indicating fetal trisomy 21. Both were confirmed by karyotyping after amniocentesis; thus, the FPR in the prospective part of the study was 0%. One blood sample represented monochorionic twins with concordant karyotypes (47,XY,+21) and the other one represented dichorionic twins with discordant karyotypes (47,XY,+21 and 46,XX). In both samples, the fetal fraction was as high as 18.0% and 24.8%, respectively. All other NIPT results were negative for trisomies 21, 18 and 13. There has been evidence of false-negative NIPT results so far in the pregnancies included in this study. Nevertheless, a number of pregnancies are ongoing (with the last birth of the patients expected in mid-May 2014) and therefore, the final detection rate is still uncertain.

**Table 2:** Characteristics and NIPT results for the prospectively collected blood samples.

Sample	Chr13 *z*-score	Chr18 *z*-score	Chr21 *z*-score	Fetal fraction (%)	Gestational age (p.m.)	No. of fetuses, chorionicity, amnionicity	NIPT result
LCMPC01	0.8	−0.4	1.0	n.a.	11 + 0	2, monochorionic, n.a.	Negative
LCMPC02	0.0	0.3	0.2	n.a.	21 + 0	2, dichorionic, diamniotic	Negative
LCMPC03	0.4	1.0	0.1	n.a.	22 + 0	2, dichorionic, diamniotic	negative
LCMPC04	−0.3	−0.6	0.0	n.a.	n.a.	3, n.a., n.a.	negative
LCMPC05	1.3	−1.0	−0.8	16.7	11 + 5	3, trichorionic, triamniotic	negative
LCMPC06	−0.4	1.1	8.5	18.0	13 + 2	2, monochorionic, n.a.	T21 positive
LCMPC07	−1.0	0.3	0.9	7.9	19 + 0	2, dichorionic, diamniotic	negative
LCMPC08	0.7	1.2	0.0	16.5	18 + 1	2, dichorionic, diamniotic	negative
LCMPC09	0.6	−0.8	0.7	8.9	11 + 5	2, monochorionic, diamniotic	negative
LCMPC10	0.3	0.7	−0.7	17.6	20 + 4	2, dichorionic, diamniotic	negative
LCMPC11	−0.9	−0.8	0.7	11.5	23 + 0	2, dichorionic, diamniotic	negative
LCMPC12	−0.9	−0.7	−2.0	13.3	11 + 1	2, monochorionic, diamniotic	negative
LCMPC13	1.3	0.1	0.3	21.4	16 + 0	2, dichorionic, diamniotic	negative
LCMPC14	0.2	−0.3	0.0	6.8	12 + 5	2, n.a., n.a.	negative
LCMPC15	2.2	0.1	14.7	24.8	16 + 0	2, dichorionic, diamniotic	T21 positive
LCMPC16	1.1	1.7	0.5	5.4	12 + 5	2, n.a., n.a.	negative
LCMPC17	0.7	1.4	0.5	16.5	14 + 2	2, n.a., n.a.	negative
LCMPC18	0.3	2.6	0.0	18.5	18 + 3	2, n.a., n.a.	negative
LCMPC19	−0.2	0.8	0.3	16.6	14 + 0	2, dichorionic, diamniotic	negative
LCMPC20	−0.7	−0.9	0.1	13.1	15 + 4	2, dichorionic, diamniotic	negative
LCMPC21	1.0	−0.7	1.2	8.4	9 + 3	2, dichorionic, diamniotic	negative
LCMPC22	−1.1	−0.2	0.3	5.6	16 + 2	2, monochorionic, n.a.	negative
LCMPC23	−2.2	2.2	−0.8	20.6	19 + 5	2, monochorionic, n.a.	negative
LCMPC24	−1.6	−0.4	−0.5	14.7	22 + 2	2, monochorionic, diamniotic	negative
LCMPC25	−0.8	−0.2	−1.5	12.1	11 + 5	2, n.a., n.a.	negative
LCMPC26	−0.4	−0.6	−1.3	7.5	13 + 0	2, dichorionic, diamniotic	negative
LCMPC27	0.5	−0.8	−0.4	16.3	12 + 6	2, n.a., n.a.	negative
LCMPC28	−1.2	−0.3	−0.7	19.4	10 + 1	2, dichorionic, diamniotic	negative
LCMPC29	−0.8	0.7	−0.4	14.2	13 + 2	2, monochorionic, n.a.	negative
LCMPC30	0.7	0.3	0.9	14.9	12 + 2	2, monochorionic, monoamniotic	negative
LCMPC31	−0.2	0.3	−0.9	19.3	19 + 1	2, dichorionic, diamniotic	negative
LCMPC32	−1.1	2.5	−2.2	11.6	20 + 0	2, dichorionic, diamniotic	negative
LCMPC33	0.2	2.2	−1.6	8.6	11 + 0	2, dichorionic, diamniotic	negative
LCMPC34	−1.0	1.2	0.0	15.1	15 + 4	2, dichorionic, diamniotic	negative
LCMPC35	−0.3	−0.8	−0.3	19.2	12 + 0	2, dichorionic, diamniotic	negative
LCMPC36	−1.4	−0.5	−0.8	13.9	12 + 0	2, dichorionic, diamniotic	negative
LCMPC37	1.8	−0.7	0.1	13.8	17 + 6	2, dichorionic, diamniotic	negative
LCMPC38	−0.1	1.1	−0.7	13.4	13 + 1	2, dichorionic, diamniotic	negative
LCMPC39	−1.9	0.2	−2.2	15.0	17 + 0	2, dichorionic, diamniotic	negative
LCMPC40	0.6	−0.4	0.8	16.2	18 + 3	2, dichorionic, diamniotic	negative

The sample collection comprised two pregnancies with triplets; both of which exhibited inconspicuous NIPT results and in both cases the newborns were reported to be phenotypically normal. To date, there are only limited data available for triplet pregnancies. To our knowledge, apart from this study, only Canick *et al*. [8] included two triplet pregnancies, both euploid. Therefore, it is still necessary to collect data before applying routine NIPT for triplets.

### 3.3. Minimum Fetal Fraction Needed for the Detection of Aneuploidies in Multiple Pregnancies

**Figure 1:**
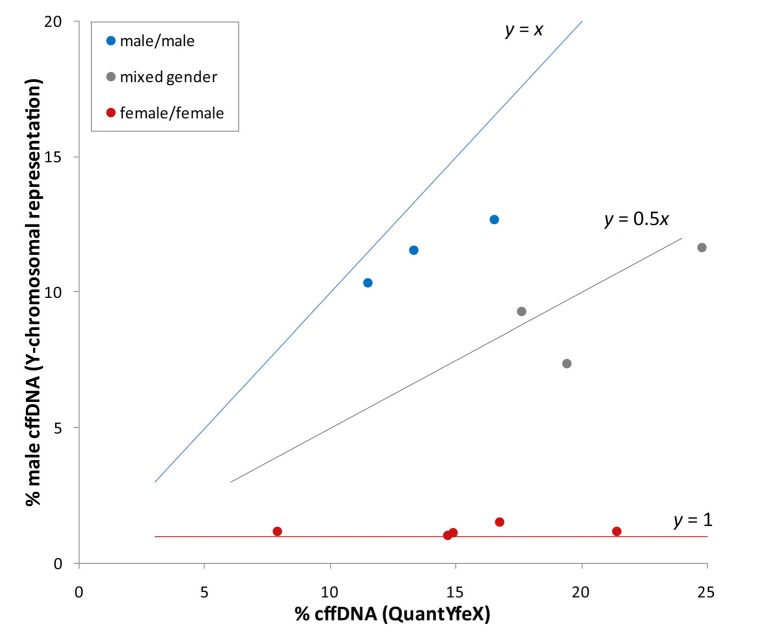
Correlation of the amount of male specific DNA to the fetal fraction measured by QuantYfeX for study cases with known fetal genders.

The reliable detection of fetal aneuploidy in twin pregnancies by NIPT is dependent on a sufficiently high amount of fetal DNA from each fetus in the maternal blood. Different data and considerations have been published on how the lower limit of cffDNA should be defined to ensure that each twin’s contribution is above the detection threshold [10,15,16]. This is especially important for dichorionic twin pregnancies with discordant karyotypes. In our study, we used supporting information for the definition of the minimum fetal fraction for twin pregnancies derived from the Y-chromosomal representation, if only one of the two fetuses is male. Using the QuantYfeX assay, the total fetal fraction can be determined, which reflects the summary of cffDNA derived from both fetuses. Using the Y-chromosomal representation from the next generation sequencing, the cffDNA amount can be determined for male fetuses (as described in [14]). Thus, in the case of mixed fetal gender, the contributing amount of each fetus can be determined by subtraction of the amount of cffDNA determined by the Y-chromosomal representation from the fetal fraction measured by QuantYfeX. We compared the fetal fractions determined by QuantYfeX with the values obtained from Y-chromosomal reads from next generation sequencing for cases with known gender (see Figure 1). There is a correlation of the amount of male specific DNA (*y*) to the fetal fraction measured by QuantYfeX (*x*). Thus, for twin pregnancies with male/male gender (*y* = *x*) is approximately true, for female/male genders it is (*y* = 0.5*x*), and for female/female (*y* = 1). The genders of cases with similar values are male/male and in case of differing values with low Y-chromosomal representation the genders are female/female. The intermediate cases, which show about half the percentage of fetal fraction as Y-chromosomal representation, are of mixed gender. On the one hand, the data presented in Figure 1 show that using this calculation it is possible to determine the fetal genders using NIPT results for twin pregnancies, although the reliability of gender determination using this combination of methylation dependent and Y-chromosomal cffDNA measurement needs to be investigated further. On the other hand, the data allow the assumption that each fetus of a twin pregnancy contributes roughly about half of the total fetal fraction. This leads to the consideration that for twin pregnancies, twice the amount of cell-free fetal DNA is necessary, and thus the minimum fetal fraction for NIPT of a twin pregnancy is 8%.

For monochorionic twin pregnancies with concordant genotypes (apart from rare exceptions [17]), a fetal fraction of 4% would be enough to detect a fetal aneuploidy, just as for single pregnancies. However, for routine laboratory NIPT service, one major issue speaks against the implication of such different quality criteria for mono- and dichorionic pregnancies: the determination of chorionicity is dependent on the gestational age and the practical experience of the physician performing the ultrasound examination. The chorionicity is clearly detectable in the first trimester of a multiple pregnancy, but in later stages, detection becomes more difficult [18]. Therefore, it is a safer strategy to generally define a minimum fetal fraction for twin pregnancies, which is applicable for monochorionic as well as for dichorionic multiple pregnancies. By targeting single nucleotide polymorphisms using massively parallel sequencing, the determination of zygosity is, in principle, possible [19] but this requires high sequencing coverage and is not, or not yet, applied in routine NIPT for multiple pregnancies.

Of the samples presented in Table 2, five contained below 8% fetal DNA and thus would be excluded in routine procedures. For four cases, the amount of fetal DNA was not available since the QuantYfeX assay was not yet available at the time of analysis. It will be interesting to see the actual failure rate in laboratory routine, as the failure rate due to a too low fetal fraction in laboratory routine for single pregnancies currently is only about 1% [20].

### 3.4. Case Reports of Discordant NIPT Results Due to Vanishing Twins

**Case report A:** An infertile couple (maternal age 39 years) underwent ART with ICSI. Following transfer of two embryos, a twin pregnancy with two gestational sacs and heart beats of both embryos were confirmed. At week 10, the heart beat for one fetus of the twin pregnancy was missing in a routine ultrasound scan. Nuchal translucency (NT) measurement at 11 weeks of gestation (p.m.) showed a normal NT (2.5 mm) for the living twin and an NT of 3.1 mm for the deceased twin. At 17 + 2 weeks of gestation, the vanishing twin was still visible in ultrasound but NIPT was performed anyway with “advanced maternal age” as the indication given. The result was positive for trisomy 21 (*z*-score 13.5, see Table 3, VTA01). Follow-up by amniocentesis revealed a discordant result for the viable child: the karyotype was 46,XY. The results from the back-up blood sample, analyzed after the discordant karyotyping result, were comparable to the first NIPT results (Table 3, VTA02) and thus confirmed the positive trisomy 21 result. For both blood samples, it was striking that the total fetal fraction measured by QuantYfeX was 20.7% and 24.8%, respectively, whereas the cffDNA according to the Y-chromosomal representation from next generation sequencing was 9.2% and 9.3%, respectively.

**Table 3:** Data for the case reports for two pregnancies with vanishing twins, which caused discordant NIPT results not representing the ongoing singleton pregnancy.

Sample code	Sample type	Gestational age	Total reads (×10^6^)	Chr13 *z*-score	Chr18 *z*-score	Chr21 *z*-score	% cffDNA calculation by ChromRep Y	Fetal fraction (QuantYfeX)
VTA01	maternal plasma sample	17 + 2	14.94	1.3	−1.5	13.5	9.2	20.7
VTA02	Back-up sample	17 + 2	17.08	0.4	−1.7	11.1	9.3	24.8
VTA03	maternal plasma sample collected prior birth	38 + 2	17.76	0.8	1.9	−0.3	21.7	21.4
VTB01	initial maternal plasma sample	13 + 2	16.79	−0.2	0.1	3.4	3.0	13.4
VTB02	Back-up sample	13 + 2	11.97	−0.1	0.3	2.6	2.7	10.0

A further blood sample taken in the third trimester of the pregnancy (38 + 2) turned out to be negative for trisomy 21 and the cffDNA amount measured by Y-chromosomal representation correlated with the fetal amount measured by QuantYfeX (21.7% and 21.4%, Table 3, VTA3), which matched the male gender determined by karyotyping the living fetus. At birth, a fetus papyraceus was found in the placental tissue from which a sufficient amount of cells could be isolated for cell culture, and following GTG banding, a trisomy 21 positive, female karyotype was confirmed (47,XX,+21).

The living twin was born phenotypically normal. For further clarification and exclusion of mosaicism material from the placenta (three different biopsies), fetal cord blood of the surviving twin as well as peripheral blood from the mother were collected. Conventional cytogenetic analyses of maternal lymphocyte cultures showed after analysis of 30 GTG banded metaphases a euploid female karyotype (46,XX). Interphase FISH analyses with FISH probes XA 21, X and Y (Metasystems) on a direct preparation of peripheral lymphocytes (*n* = 100) confirmed a normal female without evidence for maternal trisomy 21 mosaicism. Conventional cytogenetic analyses of lymphocyte cultures from the surviving twin showed after analysis of 30 GTG banded metaphases a euploid male karyotype (46,XY). Interphase FISH analyses with FISH probes XA 21, X and Y (Metasystems) on a direct preparation of peripheral lymphocytes (*n* = 100) confirmed a normal male without evidence for trisomy 21 mosaicism. Conventional cytogenetic analyses of chorion long-term cultures (mesenchymal core) from the surviving twin were also performed. Analysis of 20 GTG banded metaphases of three different biopsies showed a euploid male karyotype 46,XY in all samples. Interphase FISH analyses with FISH probes XA 21, X and Y (Metasystems) on direct preparations of each sample (*n* = 100) confirmed a normal male without evidence for trisomy 21 mosaicism. In summary, there was no evidence for a trisomy 21 mosaicism, either in the mother or in the surviving child. In summary, these results clearly indicate, that the initial NIPT result (VTA01/VTA02) represented the trisomic cffDNA of the vanishing twin. The discrepancy of the cffDNA measurement by QuantYfeX and by Y-chromosomal representation in the initial sample and the back-up sample can be explained by the presence of the deceased female fetus and the living male fetus, both releasing cffDNA into the mother’s circulation.

**Case Report B:** Routine NIPT on a maternal blood sample from gestational week 13 + 2 was performed due to an increased risk for aneuploidy based on first trimester screening in January 2014. The results of the initial sample indicated a positive result for trisomy 21 (*z*-score ≥ 3) with a *z*-score of 3.4 (Table 3, VTB01). However, as this value falls into a defined borderline range (*z*-score 2.5–3.5) the analysis was repeated independently with the back-up sample (VTB02) and the results were comparable with a *z*-score for chromosome 21 of 2.6, still being in the borderline range. For both samples, a slightly increased Y-chromosomal representation was monitored indicating male specific cffDNA of 3.0% and 2.7%, respectively. As the fetal fractions for VTB01 and VTB02 measured by QuantYfeX were far above what (13.4% and 10.0%) we hypothesized, from this discrepancy in the fetal fraction measured, we found that two fetuses with discordant gender contribute to the fetal fraction with the male fetus being the one affected by trisomy 21. This suggestion was derived from the correlation of Y-chromosome specific cffDNA-amount of roughly 3% with the elevated *z*-score around the cut-off value of 3.0. The strong correlation of trisomy 21 positive *z*-score to the cffDNA amount is already described by Palomaki *et al.* [6] and is routinely observed in our NIPT laboratory procedure. Since the examination was clearly requested for a singleton pregnancy, the male specific cffDNA was suspected to stem from a vanishing twin—maybe the carrier of a trisomy 21—that was either not recognized or not indicated on the consent form for NIPT. Thus, the result was reported to be indecisive for chromosome 21 and the conflicting data was reported to the responsible physician, including a notice regarding the potential vanishing twin, for further clarification via ultrasound. The responsible physician subsequently confirmed that the pregnancy had started as a twin pregnancy after application of ART and later continued as a singleton pregnancy. The gender of the living and apparently healthy fetus was confirmed to be female and thus, the cffDNA that caused the increased *z*-score for trisomy 21 can clearly be assigned to a deceased male fetus. The pregnancy is ongoing and further analysis of placental tissue and blood of the living fetus is not yet possible.

In summary, two cases of vanishing twins show that NIPT results should be interpreted carefully and all available data of the analysis may help to detect potential distortions of the results that may be caused by a vanishing twin. Although Futch *et al*. [21] report false-positive NIPT results which were probably caused by a deceased co-twin, there is no clinical data to date on how long the placenta of a deceased co-twin can release cell-free DNA into the maternal bloodstream. In our case A, the deceased twin did not vanish completely and the mummified fetus was present throughout the pregnancy. At the gestational age of 17 + 2, about 50% of the total fetal fraction could be assigned to the vanishing twin. At a later stage of the pregnancy (week 38 + 2), the deceased twin seemed to have stopped releasing cffDNA into the maternal circulation (see Table 3, VTA02). In case B, the vanishing or absorption procedure seemed to have nearly been finished at the point of blood sampling at week 13 + 2, since only a small proportion of total cffDNA could be assigned to the vanishing twin (about 25% of the total fetal fraction). Further studies are warranted to understand in more detail the dynamics of the vanishing or absorption process and the impact on NIPT results.

## 4. Conclusions

From the results described in this study, we conclude that NIPT is applicable for multiple pregnancies if sufficient fetal DNA of all fetuses is present in the maternal plasma. Measurement of the fetal fraction is necessary quality control for both single and multiple pregnancies. In the case of twin pregnancies, a minimum of 8% fetal DNA is required to detect common fetal trisomies, which is double the minimum amount required for singleton pregnancies (4%). NIPT results should be interpreted with care; especially for cases with a low fetal fraction and in case of multiple gestations, all available results from the analysis which support the interpretation of the results should be evaluated. For multiple pregnancies with more than two gestations, the interpretation of the results becomes even more complicated, thus further study is required to gain insight into the distribution of the proportions of cffDNA from each fetus.

Vanishing twins are a clear confounding factor for NIPT and should be carefully monitored to aid in interpreting NIPT results. It has not yet been described whether the size of a vanishing twin or the size of its amniotic cavity correlate with the amount of cell-free DNA in the maternal plasma. Furthermore, it needs to be clarified whether a vanishing twin event may lead to immediate flooding of specific cffDNA into the mother’s circulation due to dying cells causing increased release of fetal DNA and an increased duration of this process. Further documentation of the progress of vanishing twin cases in combination with NIPT results is required for a deeper understanding and clearer interpretation of results for such cases.

## Appendix

**Table A1:** PCR components.

		Component		Sequence	Concentration			Final concentration (μM)	1× μL
		master mix			2×			1×	12.5
*RASSF1* sens/insens		primer for p0009		ATT GAG CTG CGG GAG CTG GC	100 μM			1.4	0.35
		primer re p0010		TGC CGT GTG GGG TTG CAC	100 μM			1.4	0.35
		probe s0001		FAM-ACC CGG CTG GAG CGT-MGB	100 μM			0.14	0.035
		primer for p0003		GGT CAT CCA CCA CCA AGA AC	100 μM			1.4	0.35
		primer re p0004		TGC CCA AGG ATG CTG TCA AG	100 μM			1.4	0.35
		probe s0002		VIC-GGG CCT CAA TGA CTT CAC GT-MGB	100 μM			0.14	0.035
*TBX3* sens/insens		primer for p0011		GGT GCG AAC TCC TCT TTG TC	100 μM			1.4	0.35
		primer re p0012		TTA ATC ACC CAG CGC ATG GC	100 μM			1.4	0.35
		probe s0010		6FAM-CCC TCC CGG TGG GTG ATA AA-MGBNFQ	100 μM			0.14	0.035
		Primer for p0021		TGT TCA CTG GAG GAC TCA TC	100 μM			1.4	0.35
		primer re p0022		CAG TCC ATG AGG GTG TTT G	100 μM			1.4	0.35
		probe s0011		VIC-GAG GTC CCA TTC TCC TTT-MGBNFQ	100 μM			0.14	0.035
general reagents		DMSO					100%	0.025	0.625
		MgCl2					50 mM	2	1
		template							10
		water							-
		summary							25

**Table A2:** Cycler profile.

	Temperature	Time	Cycles	Analysis mode
pre-incubation	95 °C	5 min	1	none
denaturation	95 °C	10 s	45	quantification
annealing	60 °C	10 s		none
elongation	72 °C	8 s		single
cooling	40 °C			none
