# Correction: Grömminger, S., *et al.* Fetal Aneuploidy Detection by Cell-Free DNA Sequencing for Multiple Pregnancies and Quality Issues with Vanishing Twins. *J. Clin. Med.* 2014, *3*, 679-692

**DOI:** 10.3390/jcm3041333

**Published:** 2014-11-24

**Authors:** Sebastian Grömminger, Erbil Yagmur, Sanli Erkan, Sándor Nagy, Ulrike Schöck, Joachim Bonnet, Patricia Smerdka, Mathias Ehrich, Rolf-Dieter Wegner, Wera Hofmann, Markus Stumm

**Affiliations:** 1 LifeCodexx AG, Konstanz 78315, Germany; E-Mails: u.schoeck@lifecodexx.com (U.S.); j.bonnet@lifecodexx.com (J.B.); p.smerdka@lifecodexx.com (P.S.); w.hofmann@lifecodexx.com (W.H.); 2 Bahceci IVF Center, Istanbul 34330, Turkey; E-Mail: erbilyagmur@hotmail.com; 3 BioGen Medical Instruments Co. Ltd., Istanbul 34235, Turkey; E-Mail: sanlierkan@biogen.com.tr; 4 Petz Aladár Country Teaching Hospital, Győr 9023, Hungary; E-Mail: nagysandor@gyor.net; 5 Sequenom Center for Molecular Medicine, San Diego, CA 92121, USA; E-Mail: mehrich@sequenom.com; 6 Center for Prenatal Diagnosis and Human Genetics Kudamm-199, Berlin 10719, Germany; E-Mails: wegner@kudamm-199.de (R.-D.W.); stumm@kudamm-199.de (M.S.)

The authors wish to make the following corrections to this paper [1]:

On page 683 at the end of Section 3.2. lines 13–14, the word “no” is missing. 

The correct sentence should be:

“There has been **no** evidence of false-negative NIPT results so far in the pregnancies included in this study.”

The authors would like to apologize for any inconvenience caused to the readers by these changes.
